# Molecular characterization and antiviral effects of canine interferon regulatory factor 1 (CaIRF1)

**DOI:** 10.1186/s12917-022-03539-3

**Published:** 2022-12-16

**Authors:** Xiangqi Hao, Hui Chen, Yanchao Li, Bo Chen, Weifeng Liang, Xiangyu Xiao, Pei Zhou, Shoujun Li

**Affiliations:** 1 College of Veterinary Medicine, South China Agricultural University, Guangzhou, Guangdong Province, 510642 People’s Republic of China; 2 Guangdong Provincial Key Laboratory of Prevention and Control for Severe Clinical Animal Diseases, Guangzhou, Guangdong Province, 510642 People’s Republic of China; 3 Guangdong Provincial Pet Engineering Technology Research Center, Guangzhou, Guangdong Province, 510642 People’s Republic of China

**Keywords:** Canine interferon regulatory factor 1, ISGs, ISRE, Antiviral characterization, CPV-2

## Abstract

**Background:**

Interferon regulatory factor 1 (IRF1) is an important transcription factor that activates the type I interferon (IFN-I) response and plays a vital role in the antiviral immune response. Although IRF1 has been identified in several mammals, little information related to its function in canines has been described.

**Results:**

In this study, canine IRF1 (CaIRF1) was cloned. After a series of bioinformatics analyses, we found that the CaIRF1 protein structure was similar to that of other animal IRF1 proteins, including a conserved DNA-binding domain (DBD), an IRF-association domain 2 (IAD2) domain and two nuclear localization signals (NLSs). An indirect immunofluorescence assay (IFA) revealed that CaIRF1 was mainly distributed in the nucleus. Overexpression of CaIRF1 in Madin-Darby canine kidney cells (MDCK) induced high levels of interferon β (IFNβ) and IFN-stimulated response element (ISRE) promoter activation and induced interferon-stimulated gene (ISG) expression. Subsequently, we assayed the antiviral activity of CaIRF1 against vesicular stomatitis virus (VSV) and canine parvovirus type-2 (CPV-2) in MDCK cells. Overexpression of CaIRF1 effectively inhibited the viral yields of VSV and CPV-2, while knocking down of CaIRF1 expression mildly increased viral gene copies.

**Conclusions:**

CaIRF1 is involved in the cellular IFN-I signaling pathway and plays an important role in the antiviral response.

**Supplementary Information:**

The online version contains supplementary material available at 10.1186/s12917-022-03539-3.

## Background

The innate immune response is recognized as the first line of defense in an organism. Interferon is a multifunctional cytokine with immune-enhancing, broad-spectrum antiviral biological activity. When responding to different viral infections, type I interferon (IFN-I) can be produced in most cells [1]. Activated IFN-I can stimulate the activation of interferon-stimulated response elements (ISREs) and promote the expression of interferon-stimulated genes (ISGs), leading to antiviral effects. Interferon regulatory factors (IRFs) constitute a family of transcription factors whose main function is the regulation of interferon production [2]. To date, 11 IRFs have been identified [3]. Among these IRFs, IRF1 is one of the important regulators that controls the transcription of IFN-I genes. IRF1 can bind not only the IFNβ promoter to activate IFN-I pathway but also ISRE directly to stimulate the expression of ISGs, inducing IFN-I pathway activity effectively [4–6]. IRF1 is an essential regulator of the IFN-I and JAK-STAT signaling pathways. The unique function of IRF1 ensures the formation of a positive feedback loop for IFN-I signaling, thus exerting an antiviral function [7].

As an important regulator, the activity of IRF1 in mammals (humans, pigs, etc), fishes, and birds have been described [5, 8–10]. However, no studies have elucidated the role played by IRF1 in canines. In this study, we cloned CaIRF1 from canine peripheral blood lymphocytes (CPBLs) and analyzed its structural characteristics. Additionally, the role played by CaIRF1 in the canine IFN-I signaling pathway and its antiviral function were identified. This study demonstrates that CaIRF1 activates the response of IFN-I and ISREs and shows anti-canine parvovirus type 2 virus (CPV-2) and anti-vesicular stomatitis virus (VSV) activity in vitro, which indicates that the function of CaIRF1 is evolutionarily conserved [11].

## Results

### Sequence analysis of CaIRF1

**Fig. 1:**
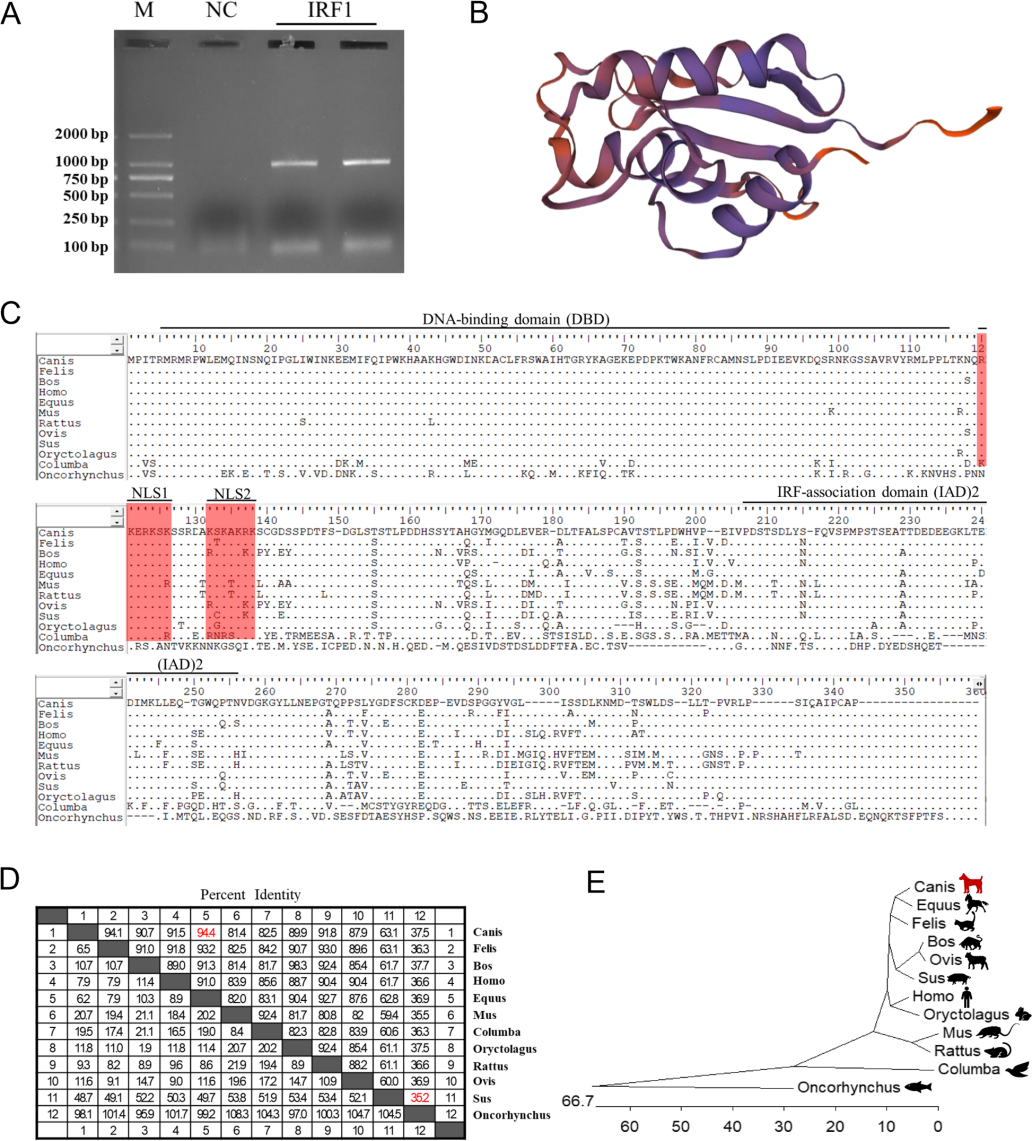
Bioinformatics analysis of CaIRF1. **A** PCR amplification of CaIRF1. M, DNA marker; NC, negative control. **B** Prediction of the 3D structure of CaIRF1 (based on SWISS-MODEL). **C** Sequence and structural domains of the IRF1 protein. The DBD and IAD2 are marked with black lines; the NLSs are labeled with a red background. **D** Homology analysis of CaIRF1 among species. **E** Phylogenetic tree analysis of IRF1 amino acids

The CDS of CaIRF1 was cloned from CPBLs obtained from a beagle and uploaded to GenBank (accession number: MW748175), which showed 100% similarity to a previously predicted sequence. The length of the CaIRF1 CDS is 996 bp (Fig. 1A), and the CDS encodes a protein containing 321 amino acids. The structure of CaIRF1 was predicted using SWISS-MODEL, and CaIRF1 was found to be mainly composed of α-helices and β-folds (Fig. 1B). In addition, multiple alignments of the amino acid sequences were run to more thoroughly investigate the similarity of CaIRF1 to IRF1 sequences in other animals. The DNA-binding domain (DBD) in the N-terminal of CaIRF1, IRF-association domain 2 (IAD2) in the C-terminal and a nuclear localization signal (NLS) in the middle of the sequence were found to be conserved on the basis of a comparison analysis (Fig. 1C). The amino acid sequence of CaIRF1 showed the highest homology with equine IRF1 at 94.4%, with *Homo sapiens* at 91.5% and with Oncorhynchus at only 37.5% (Fig. 1D). Phylogenetic analysis showed that CaIRF1 clustered with mammalian IRF1 in a large branch (Fig. 1E). These results indicated that the sequence of CaIRF1 was evolutionarily conserved.

### CaIRF1 activates IFNβ and ISRE promoters

**Fig. 2:**
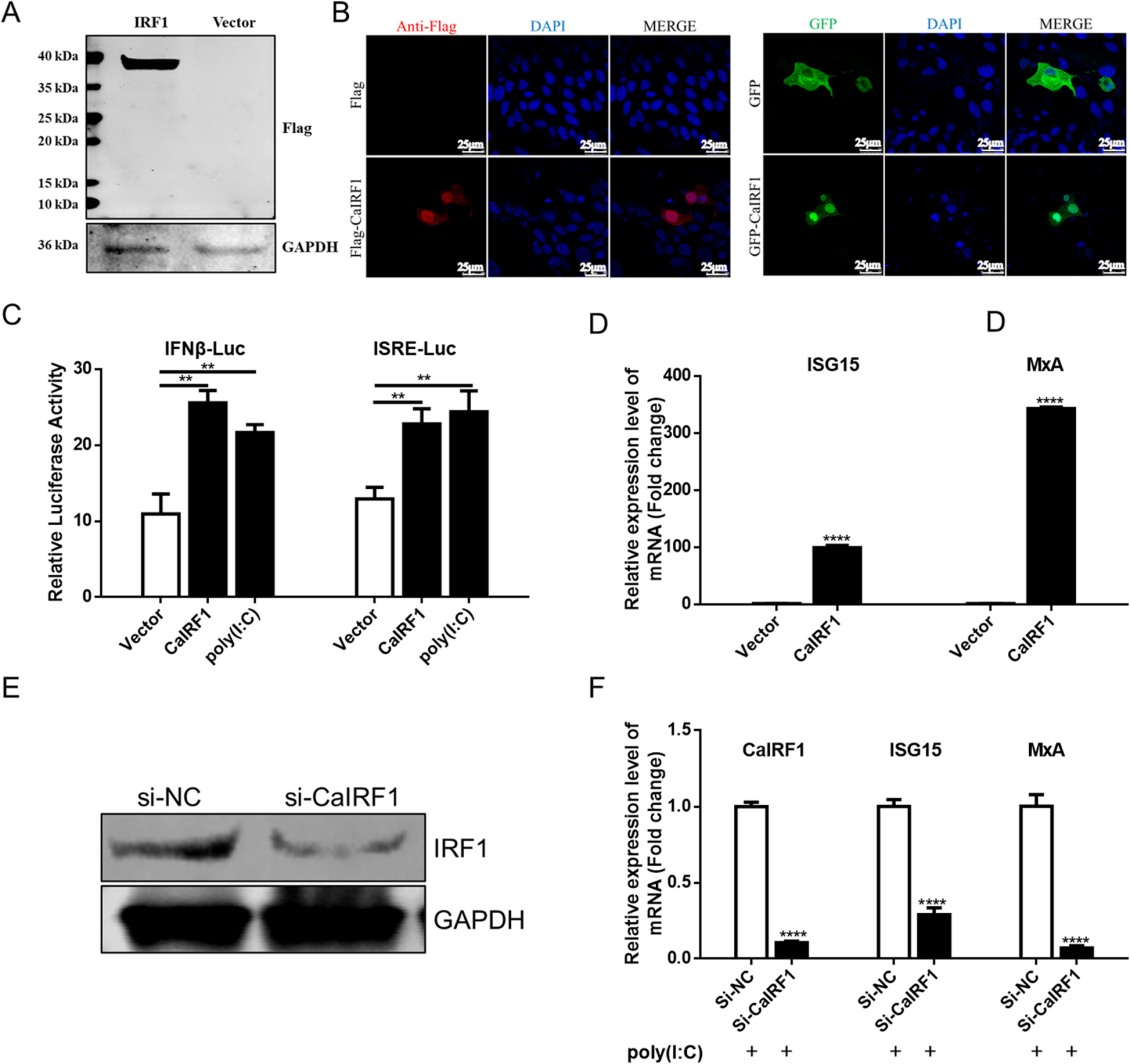
CaIRF1 activates the IFNβ and ISRE promoters. **A** Western blot to determine CaIRF1 expression. p3 × Flag-CaIRF1 (0.5 μg) or empty plasmid (0.5 μg) was transfected into cells cultured on 24-well cell plates, and 24 h later, western blot was carried out to verify the expression of CaIRF1. GAPDH was used as the loading control. **B** IFA and GFP tag observation verified that CaIRF1 was expressed and localized mainly in the nucleus. Adjustments of individual colour channels are sometimes necessary on merged images. This part of the experiment was photographed under a confocal microscope (LEICA, TSC-SP8). Scale bar, 25 μm. **C** A dual-luciferase assay was performed to detect the activation of IFNβ and ISRE promoters induced by CaIRF1. The specified vector (0.5 μg) was cotransfected with the promoter plasmid (0.5 μg) and pRL-TK (0.025 μg). Twenty-four hours post-transfection, cells were stimulated with the poly(I:C) for 12 h or with no treatment and then assayed for IFNβ and ISRE promoter activity. **D** Results showing the effect of CaIRF1 regulation on the expression of ISG mRNAs (ISG15 and MxA). **E** MDCK cells were transfected with si-NC or si-CaIRF1 (40 pmol) for 36 h. Finally, the expression of endogenous CaIRF1 was measured by western blot. GAPDH was used as a loading control. **F** Knocking down CaIRF1 blocked the poly(I:C)-induced expression of ISGs. MDCK cells were transfected with si-NC or si-CaIRF1 (40 pmol) for 36 h. The cells were stimulated with the addition of poly(I:C) for 12 h, and then, the transcription of ISGs was detected by qPCR. The data shown in the figure represent the mean ± SD of three independent experiments, each experiment had three cell wells as technical replicates. ** *P* < 0.01, **** *P* < 0.0001 compared with the empty vector transfection group. The original images of the western blot and enlarged versions of the cell images were shown in the Supplementary file 1

Indirect immunofluorescence assay (IFA) and western blot were carried out to determine whether CaIRF1 plasmids were expressed in Madin-Darby canine kidney (MDCK) cells. The results showed that the molecular weight of the Flag-CaIRF1 protein was approximately 40 kDa (Fig. 2A), which was consistent with the predicted results. Through confocal microscopy, CaIRF1 was found to be mainly distributed in the nucleus (Fig. 2B), which may be related to CaIRF1 carrying two NLSs (Fig. 1C).

The IFN-I and ISG responses are key steps in antiviral innate immunity. To determine whether CaIRF1 induces activation of the IFNβ and ISRE promoters, p3 × Flag-CaIRF1 was cotransfected with pIFNβ-Luc or pISRE-Luc in MDCK cells. The poly(I:C) and vector groups were the positive control and negative control, respectively, and luciferase activity was analyzed 24 h after transfection. The results showed that overexpression of CaIRF1 caused high IFNβ and ISRE promoter activation (Fig. 2C). Second, interferon-stimulated gene 15 (ISG15) and myxovirus resistance protein A (MxA) were activated for transcription, resulting in a potential antiviral effect (Fig. 2D). To investigate whether blocking endogenous CaIRF1 expression affects the antiviral response, siRNA targeting CaIRF1 (si-CaIRF1) was transfected into MDCK cells, and nontargeting siRNA (si-NC) was used as the control. The knockdown efficiency of CaIRF1 expression was determined by western blot. The results showed that si-CaIRF1 exposure resulted in a significant decrease in endogenous CaIRF1 expression 36 h after transfection compared to expression in the control group (Fig. 2E). Subsequently, poly(I:C) was added to the cells in the CaIRF1-knockdown group and the control group to stimulate transcription, and the transcript levels of CaIRF1 and ISGs were measured after 12 h of treatment. The results showed that CaIRF1 expression was significantly decreased and that knocking down CaIRF1 expression led to blocked induction of ISG15 and MxA by poly(I:C) (Fig. 2F). Overexpression of CaIRF1 activated the IFNβ and ISRE promoters and promoted the transcription of ISGs. In contrast, knocking down CaIRF1 expression blocked the transcription of poly(I:C)-stimulated ISGs. Our results show that CaIRF1 plays an important role in the antiviral response.

### VSV and CPV-2 yields were low in MDCK-CaIRF1 cells

**Fig. 3:**
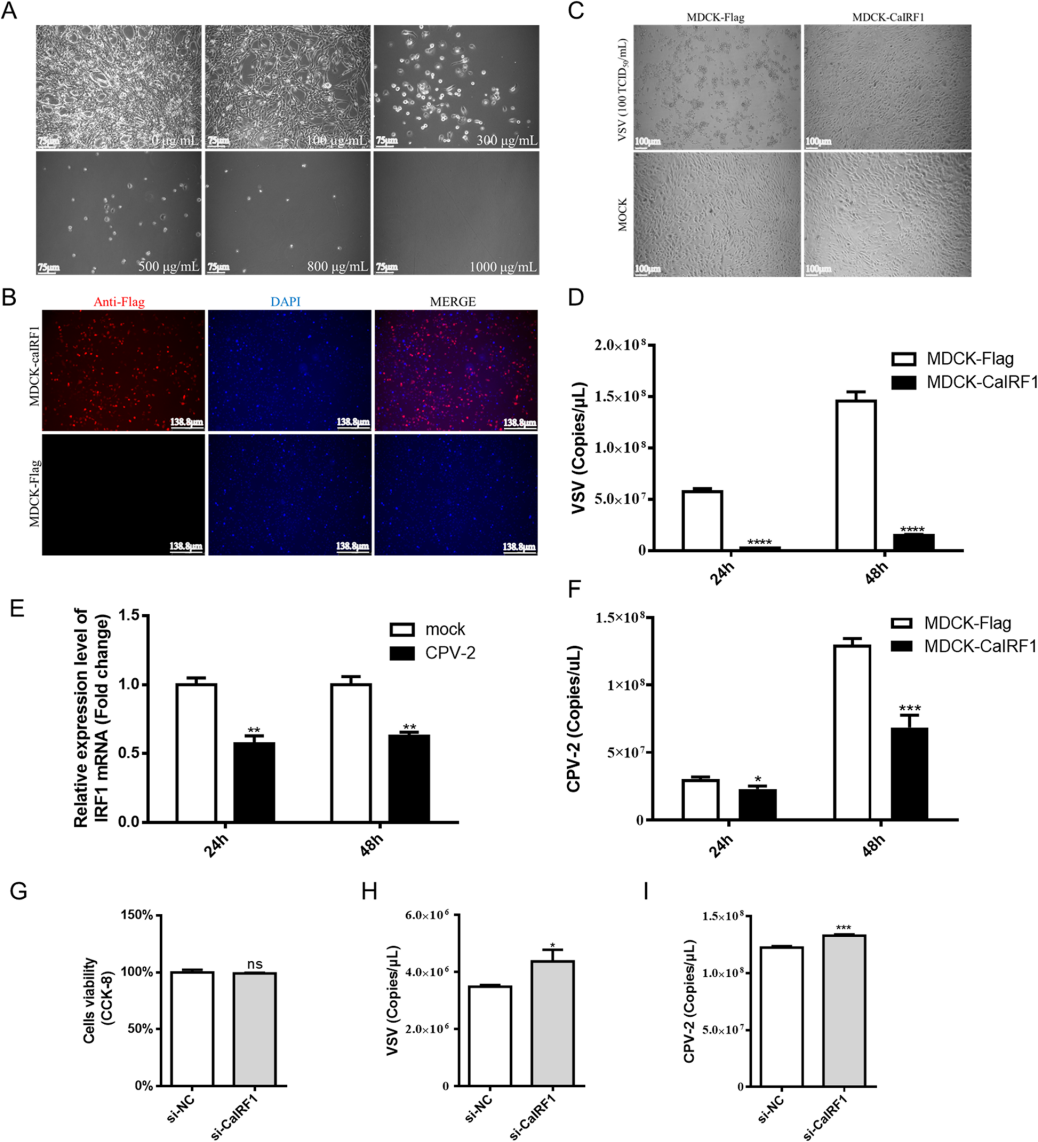
Validation of the antiviral function of CaIRF1. The cells in this part of the assay were photographed under an inverted fluorescence microscope (Leica, D35578), camera model DFC3000G, with Leica application suite X. **A** Minimum G418 concentration screen to determine the minimal necessary to kill MDCK cells. The minimum concentration used in the study was determined to be 800 μg/mL. Scale bar, 75 μm. **B** Validation of MDCK cells stably expressing CaIRF1. An IFA was performed to identify cells that stably expressed CaIRF1. Adjustments of individual colour channels are sometimes necessary on merged images. The scale bar represents 138.8 μm. **C** MDCK-CaIRF1 protected cells against cytopathogenic lesions induced by VSV. The scale bar represents 100 μm. **D** Less VSV replicated in the MDCK-CaIRF1 cells than in the MDCK cells. **E** CPV-2 infection of MDCK cells inhibited the expression of CaIRF1 mRNA. **F** CaIRF1 inhibited the replication of CPV-2 in cells. **G** A CCK-8 assay was performed to determine the viability of si-CaIRF1- and si-NC-transfected MDCK cells (36 h). **H** and **I** Slightly increased replication of CPV-2 and VSV was found in CaIRF1-knockdown cells. The data shown in the figure represent the mean ± SD of three independent experiments, each experiment had three cell wells as technical replicates. * *P* < 0.05, ** *P* < 0.01, *** *P* < 0.001, **** *P* < 0.0001 compared with the empty vector transfection group. The enlarged versions of the cell images were shown in the Supplementary file 2

To explore the antiviral effect of CaIRF1, the viral yields of VSV and CPV-2 in the MDCK-CaIRF1 cell line were evaluated. First, the MDCK-CaIRF1 cell line was obtained using the G418 screening method (Fig. 3A). The IFA demonstrated that the MDCK-CaIRF1 cell line stably overexpressed CaIRF1 (Fig. 3B).

Second, MDCK-Flag cells and MDCK-CaIRF1 cells were counted separately and seeded into 24-well plates and then incubated with VSV at an MOI of 1. Twenty-four hours post-inoculation, the MDCK-Flag cells in the control group died after developing cytopathy, whereas the MDCK-CaIRF1 cells continued to grow (Fig. 3C). A qPCR assay determined that fewer VSV viral copies were produced in the MDCK-CaIRF1 cells, indicating that CaIRF1 showed antiviral activity against VSV in vitro (Fig. 3D).

Third, according to previous studies, IFNβ exerts a small antiviral effect on parvovirus replication [12, 13]. However, few studies have reported the mechanism by which this virus regulates CaIRF1 or whether CaIRF1 inhibits CPV-2 replication. MDCK cells were seeded in 24-well plates, and CPV-2 was inoculated at an MOI of 1. CPV-2 infection inhibited the mRNA expression of CaIRF1 as determined by qPCR (Fig. 3E). In addition, the number of CPV-2 copies were measured, and the results showed that the number of CPV-2 copies in the MDCK-CaIRF1 was lower than that in the MDCK-Flag cells (Fig. 3F).

Finally, the CPV-2 and VSV viral yields were examined in cells with CaIRF1 gene expression knocked down. siRNA targeting CaIRF1 was transfected into MDCK cells, and cell viability was not affected (Fig. 3G). However, CPV-2 and VSV viral yields increased after inoculation of CaIRF1-knockdown cells (Fig. 3H&I). In conclusion, our results suggested that knocking down CaIRF1 expression moderately enhanced the production of CPV-2 and VSV in the MDCK cells.

## Discussion

The innate immune system serves is the first line of defense for the host and plays a crucial role in the recognition and clearance of pathogens [14]. IRF1 was the first member of the interferon regulatory family to be identified, and it identified through its participation in the regulation of IFN-α/β expression [15]. Studies on IRF1 have focused on humans, pigs, birds or fishes [5, 8, 9], and descriptions of the role played by CaIRF1 were insufficient. In this study, we cloned the CDS of CaIRF1 and examined its molecular characterization and antiviral effect. We found that CaIRF1 activated the IFNβ and ISRE promoters, promoted the transcription of ISGs, and effectively inhibited the production of VSV and CPV-2 in MDCK cells, demonstrating that CaIRF1 plays an antiviral role in vitro.

In this study, the CaIRF1 gene was cloned from CPBLs. The CaIRF1 protein is similar to IRF1 in other animals, with structural features that include a conserved DBD, IAD2 and two NLSs. CaIRF1 has the highest homology with equine IRF1 (94.4%). A phylogenetic tree showed that CaIRF1 and other mammalian IRF1 are in one subgroup, and the other species belong to other subgroups. On the basis of these results, we speculate that the CaIRF1 sequence is relatively conserved and that its antiviral activity also is likely similar to that in other species [5, 16, 17].

IRF1 effectively activates the IFN-I pathway. First, IRF1 binds to the IFN promoter, upregulating the expression of IFN-α/β and activating the type IFN-I pathway. In addition, the N-terminal DBD of IRF1 can directly bind to ISRE, stimulating the production of ISGs and thus an effectively activate the IFN-I pathway [4]. IRF1 not only acts as an ISG to regulate the production of IFN-I but is also an important factor that function in combination with ISRE to generate an antiviral effect mediated by the JAK-STAT signaling pathway. In this study, we proved that CaIRF1 can highly activate the IFNβ and ISRE promoters. Overexpression of CaIRF1 promotes the transcription of ISGs. In contrast, knocking down of CaIRF1 blocks poly(I:C)-stimulated expression of ISGs (ISG15 and MxA). CaIRF1 can guarantee the formation of a positive feedback loop through IFN-I signaling and thus shows versatility as a contributor to antiviral innate immunity.

Among the viruses in dogs, CPV-2 often causes severe disease, and more importantly, CPV-2c variants are growing at an alarming rate [18, 19]. In addition, VSV is an IFN-I-sensitive virus, and it is a good tool to be used for antiviral activity assessment [20] . Therefore, CPV-2 and VSV were selected for the antiviral tests. However, previous studies had shown that parvovirus infection can block the IFN-I pathway [13]. After infection, IFN-I fails to inhibit parvovirus replication [12], and we search for a novel antiviral factor, CaIRF1. Interestingly, CaIRF1 can inhibit the production of VSV and CPV-2 in MDCK cells and shows an adequate antiviral effect on MDCK cells. Although CPV-2 downregulates the mRNA expression of CaIRF1, thereby antagonizing the IFN-I pathway, overexpression of CaIRF1 effectively inhibits the replication of CPV-2. We speculate that these outcomes were related to the properties of CaIRF1, as it can both activate IFN-I transcriptional activation and directly stimulate ISGs expression [5, 21]. Even when the IFN-I signaling pathway was blocked, CaIRF1 directly bound to ISRE, thus exhibiting its antiviral function. IRF1 has a short half-life (only 30 min) and degrades rapidly after activation, inducing with negligible side effects [15].

## Conclusions

In this study, we cloned CaIRF1 and characterized its antiviral activity. CaIRF1 is an important regulator of IFN-I activity and ISG expression, which can inhibit the production of CPV-2 and VSV and play roles in antiviral innate immunity.

## Methods

### Cells and viruses

MDCK cells were purchased from iCell (Shanghai, China), cultured in Dulbecco’s modified Eagle’s medium (DMEM) with 10% fetal bovine serum (FBS) in 5% CO_2_ at 37 °C. CPBLs obtained from a healthy beagle of our laboratory were used to amplify the CaIRF1 gene. The CPV-2 and VSV used in this study were stored in our laboratory, and the TCID_50_ was measured before use. In short, the VSV or CPV-2 was diluted in a 10-fold gradient, and 0.1 ml of diluted virus was added to the 96-wells. After 2 h of viral adsorption in MDCK cells, new DMEM containing 1% FBS was added. The replication of VSV and CPV-2 was measured by observing cytopathic effect or IFA respectively after 72 h of infection and were expressed as the median log10 (TCID_50_/ml) based on the Reed and Muench method [22, 23], and referred to our previous study [24]. In the antiviral activity assay, the doses used for both VSV and CPV-2 were set at an MOI of 1.

### Plasmids and reagents

The canine promoter reporter plasmid pIFNβ-Luc was constructed according to a previous study [25]; a pISRE-Luc and pRL-TK were purchased from Beyotime (Shanghai, China) and Promega (USA), respectively. Poly(I:C) (Sigma, USA) at a final concentration of 2 g/mL was the control for promoter activation experiments. Lipo8000 (Beyotime, China) was used for plasmid transfection according to the manufacturer’s instructions. Anti-GAPDH, anti-Flag-tag mouse monoclonal antibody and anti-IRF1 rabbit monoclonal antibody (Beyotime, China) were used in IFA and western blot. Small interfering RNA (siRNA) (+) against CaIRF1 sequence, 5′-GCACCAGUGACCUCUACAGTT-3′, the siRNA (−) sequence, 5′-CUGUAGAGGUCACUGGUGCTT-3′, and the negative control (NC) siRNA were purchased from Tsingke Biotech (Beijing, China). The primers used in the study were synthesized by Sangon Biotech (Shanghai, China).

### Cloning and analysis of the CaIRF1 sequence

Primers for amplifying CaIRF1 were designed based on the predicted sequences (XM_038681484.1) in the NCBI database (https://www.ncbi.nlm.nih.gov/genbank/) (Table 1). Then, a Fastagen extraction kit was used to extract total RNA from CPBLs. First-strand cDNA was reverse transcribed and used as a template to amplify the CaIRF1 coding sequence (CDS). Next, the PCR product was ligated to the pclone007 plasmid and later transformed into the DH5α competent cell. After a screening culture, the monoclonal bacteria were selected and sent to Sangon Biotech for sequencing. Finally, the sequenced vector was digested with HindIII and BamHI, and the product was ligated to a p3 × Flag-CMV and pEGFP-C3 for expression.

**Table 1:** Primers used in this study

Primers	Sequences (5′ → 3′)	Purpose
CaIRF1-F	CCCAAGCTTATGCCCATCACTCGGATGCG	Amplification of CaIRF1
CaIRF1-R	CGCGGATCCCTATGGTGCACAAGGAATGGCCT	
q-VSV-F	ACTCAGCCTTGTGGATGACC	qPCR for detection of VSV
q-VSV-R	CCCGTGTACTCGTCCACTTT	
q-CPV-2F	CATTGGGCTTACCACCATTT	qPCR for detection of CPV-2
q-CPV-2R	AAATGGCCCTTGTGTAGACG	
q-CaGAPDH-F	CAAGAAGGTAGTGAAGCAGGCATC	qPCR for detection of CaGAPDH
q-CaGAPDH-R	TCGAAGGTGGAAGAGTGGGTG	
q-CaIRF1-F	GGAAGTGAAGGACCAGAGC	qPCR for detection of CaIRF1
q-CaIRF1-R	TCCATCAGAGAAGGTGTCA	
q-CaISG15-F	AGTATCGCCTACGAGGTCTG	qPCR for detection of CaISG15
q-CaISG15-R	ATGGGCTTCCCTTCAAAA	
q-CaMxA-F	TTGAGGACCACCCACATTTC	qPCR for detection of CaMxA
q-CaMxA-R	CAGAGGCAGGGTTTTACAGATG	

*F* forward primer, *R* reverse primer. Restriction sites are labeled using an underline

InterProScan (http://www.ebi.ac.uk:/Tools/pfa/iprscan/) was used to analyze the structural domain of CaIRF1, and SWISS-MODEL (https://swissmodel.expasy.org) was used for CaIRF1 homology modeling [26]. The IRF1 gene sequences in different species, including mammals (Bos NP_001178190.1, Felis XP_003980801.1, Homo AAA36043.1, Equus XP_005599497.1, Mus XP_021068655.1, Sus NP_001090882.1, Rattus NP_036723.1, Oryctolagus NP_001164818.1, Ovis NP_001009751.1), a bird (Columba PKK20647.1), and a fish (Oncorhynchus AAM77843.1), were downloaded from the NCBI database, followed by amino acid multiple sequence alignment with BioEdit. Finally, an evolutionary analysis was performed with DNAStar, and a phylogenetic tree was constructed.

### IFA assay

MDCK cells were inoculated in small dishes, and CaIRF1 expression plasmids and control empty vector were transfected into the cells with Lipo8000. Thirty-six hours after transfection, the cells were fixed with 4% paraformaldehyde and incubated with Triton X-100 and 5% skim milk to facilitate blocking and membrane permeabilization. Next, monoclonal anti-Flag antibody was added dropwise to the cell surface at 4 °C for 6 h. After that, cells were washed three times with PBS for 5 min each time, and then the cells were incubated with a fluorescent secondary antibody (Alexa Fluor 594 conjugated) (Cell Signaling Technology, Inc.) against mouse IgG at 4 °C overnight. Followed, washing the cells again with PBS for 3 times. Finally, anti-fluorescence attenuators (containing DAPI) (Solarbio, China) were added dropwise prior to fluorescence microscopy observation. All images were automatically captured by the software accompanying the microscope.

### Luciferase reporter assays

According to the instructions, MDCK cells grown in 48-well plates were cotransfected with IFNβ-Luc, pRL-TK, p3 × Flag-CaIRF1 or p3 × Flag empty vector using Lipo8000 (Beyotime, China). Then, poly(I:C) was added after transfection for 24 h. The cells were harvested after another 12 h of incubation and analyzed using a dual luciferase kit (Promega). The results of the dual luciferase assay from three independent biological replicates were combined and reported.

### Construction of the MDCK-CaIRF1 cell line

First, we determined the minimum concentration of G418 sulfate needed to kill MDCK cells. MDCK cells were seeded in 6-well plates, and when the growth density reached 20–25%, DMEM with G418 sulfate (at concentrations of 100, 300, 500, 800, and 1000 μg/mL) was added. Then, the G418 screening medium (containing 10% FBS and different concentrations of G418) was replaced every 3 days. To construct the cell lines, MDCK cells were transfected with the expression plasmid p3 × Flag-CMV-CaIRF1, and screening medium (800 μg/mL) was added 24 h after transfection. All the control cells died after 14 days, at which time the surviving cells in the transfected group stably expressed CaIRF1. Similarly, the control MDCK-Flag cells were obtained by screening after transfection with p3 × Flag-CMV plasmid.

### Real-time qPCR assay

Total RNA was extracted from cells with an RNA extraction kit (Fastgen, China), and the RNA concentration was measured with a Nanodrop One spectrophotometer (Thermo, USA). Total RNA (2 μg) was reverse transcribed into cDNA with an ABScript II RT Mix kit (ABclonal, China). The real-time qPCR system was configured using the appropriate primers (Table 1) with SYBR Green I Mix (ABclonal, China). GAPDH was the housekeeping gene and used to normalize the relative levels of mRNA. CPV-2 and VSV genome copy numbers were determined by referring to previously established methods [27, 28].

### Western blot

MDCK cells were seeded into 6-well plates and later transfected with expression plasmids. After 36 h, the cells were washed with PBS and lysed on ice with RIPA buffer. Then, protein samples were added to polyacrylamide gels for electrophoresis. After electrophoresis, the separated proteins were transferred to polyvinylidene fluoride membranes. The membrane was blocked with 5% bovine serum albumin (BSA) for 1 h at room temperature. Then, the membrane was washed three times with TBS for 5 min each time, after which the anti-Flag monoclonal antibody was added dropwise to the membrane surface at 4 °C for 6 h. And then the membrane was washed three times again, following, incubated with the anti-mouse IgG secondary antibody (IRDye800CW Conjugated) (LI-COR, USA). As for the detection steps, the target signal was scanned using an Odyssey near infrared membrane sweeper.

### Cell counting Kit-8 (CCK-8) assay

MDCK cells were inoculated into 96-well plates and later transfected with the corresponding siRNA, and a blank group was established. Twenty-four hours post-transfection, 10 μL of CCK-8 was added to each cell well and incubated for 2 h. The final absorbance was measured with a microplate reader at 450 nm.

### Statistical analysis

Each statistical analysis was based on 3 biological replicate experiments, and t tests were performed to determine the statistical significance of differences, with values of *P* < 0.05 considered to be statistically significant and *P* < 0.01 considered highly statistically significant. The graphs presented in the text were generated with GraphPad Prism 6 software.

## Supplementary Information

**Additional file 1.**

**Additional file 2.**

## Data Availability

The datasets used and/or analysed during the current study are available from the corresponding author on reasonable request.

## Abbreviations

IRF1: Interferon regulatory factor 1
IFN-I: Type I interferon
CaIRF1: Canine IRF1
DBD: DNA-binding domain
IAD2: IRF-association domain 2
IFA: Indirect immunofluorescence assay
NLSs: Nuclear localization signals
ISRE: IFN-stimulated response element
ISG: Interferon-stimulated gene
VSV: Vesicular stomatitis virus
CPV-2: Canine parvovirus type-2
CPBLs: Canine peripheral blood lymphocytes
DMEM: Dulbecco’s modified Eagle’s medium
NC: Negative control
CDS: Coding sequence
BSA: Bovine serum albumin
MxA: myxovirus resistance protein A
ISG15: Interferon-stimulated gene 15
