# Supplementary figures and images for: Molecular characterization and antiviral effects of canine interferon regulatory factor 1 (CaIRF1)
Source: BMC Vet Res. 2022 Dec 16;18:440. doi: 10.1186/s12917-022-03539-3 (PMC9756622; doi:10.1186/s12917-022-03539-3)

Fig. 2A Flag

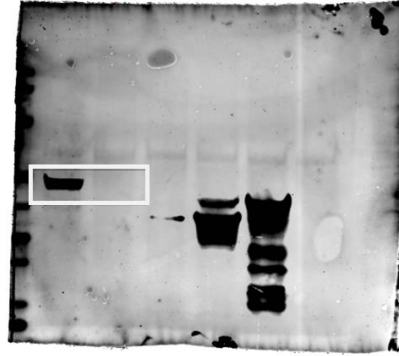

Fig. 2A GAPDH

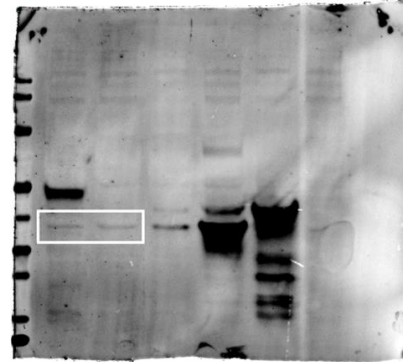

Fig. 2E IRF1

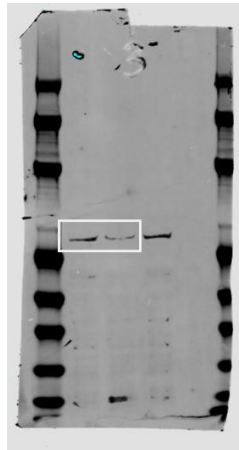

Fig. 2E GAPDH

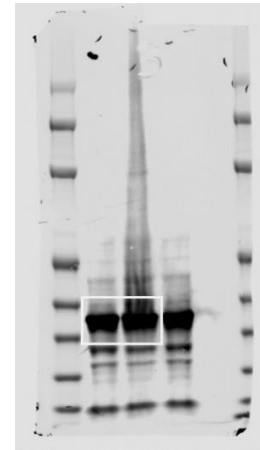

Fig. 2B

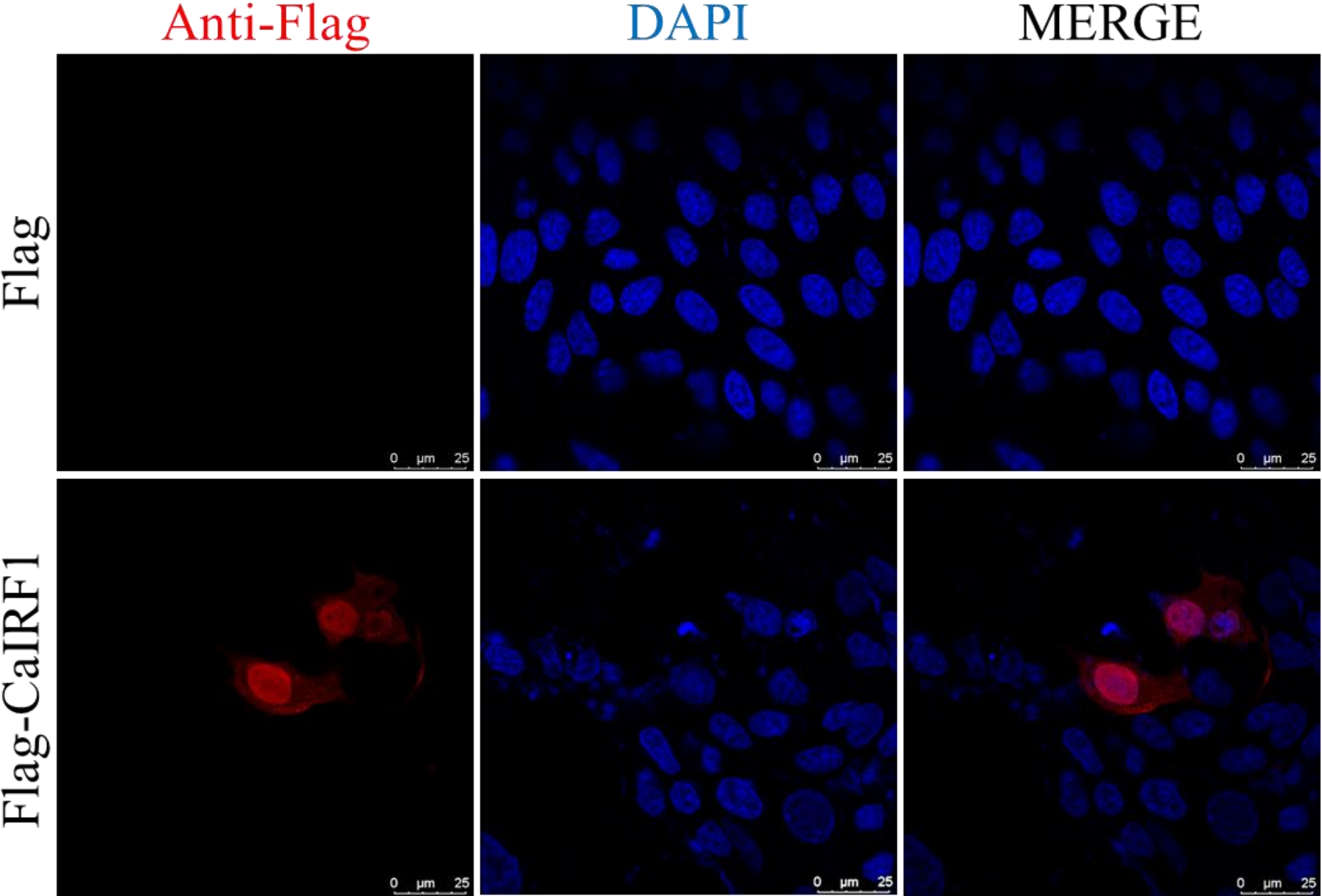

Fig. 2B

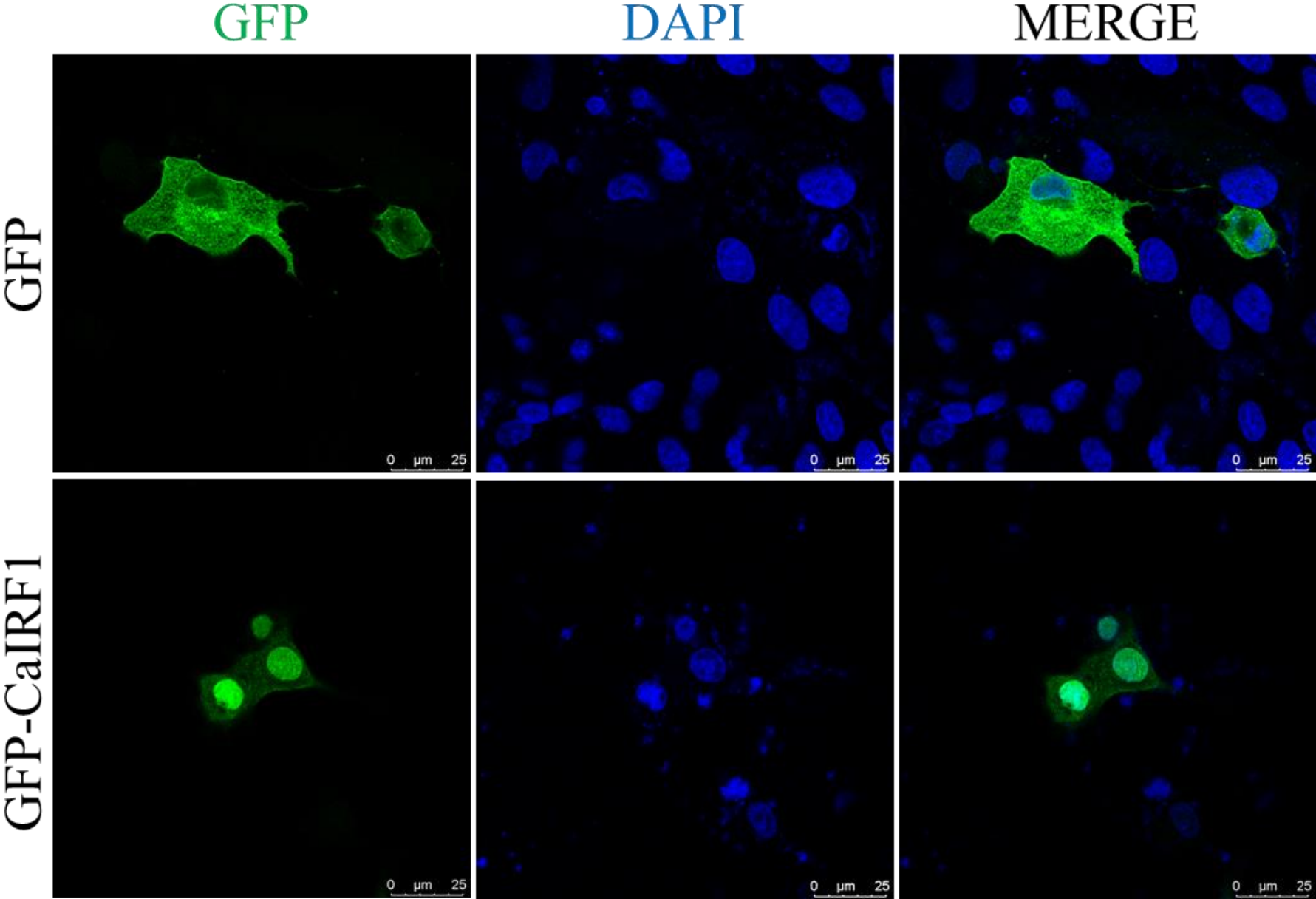

Supplement: Supplementary file 1 — Additional file 1. [file 12917_2022_3539_MOESM1_ESM.pdf]

Fig. 3A

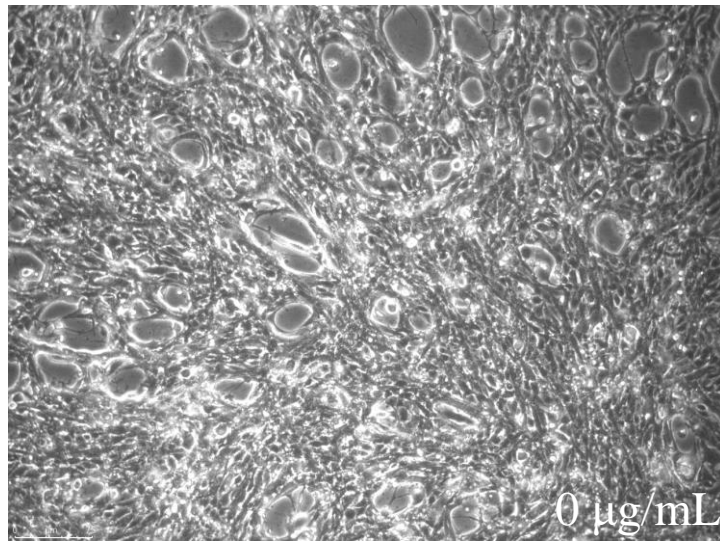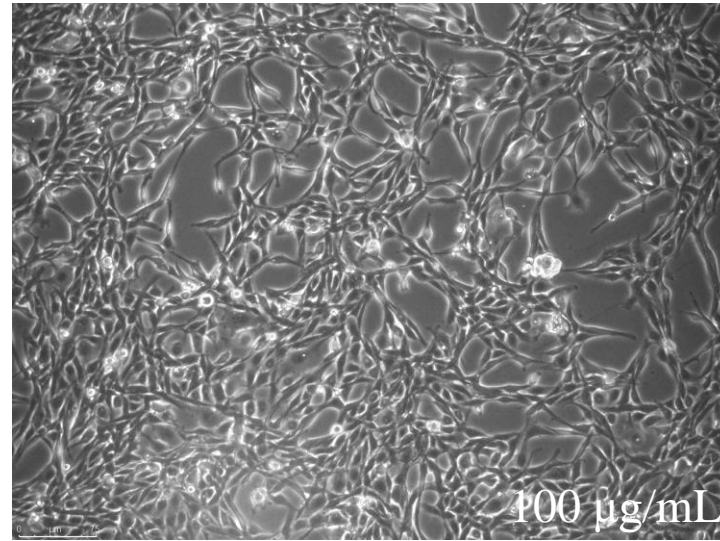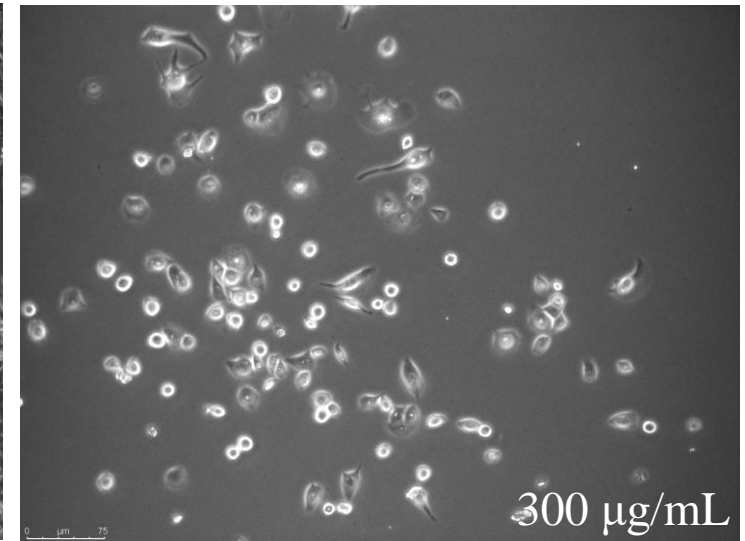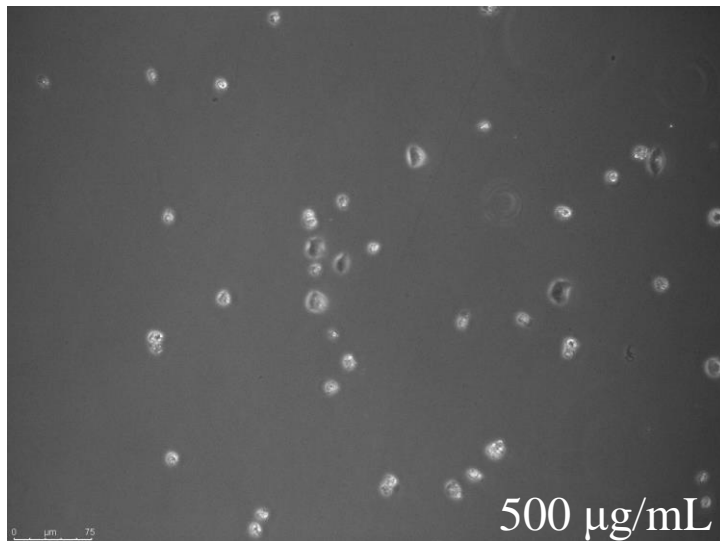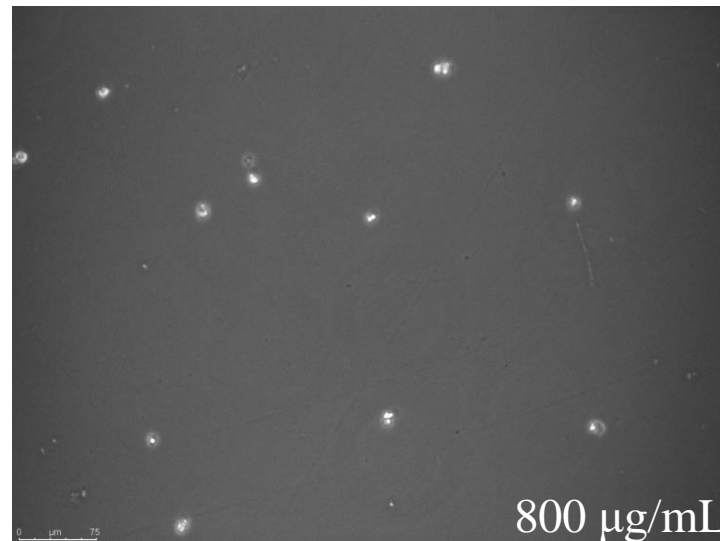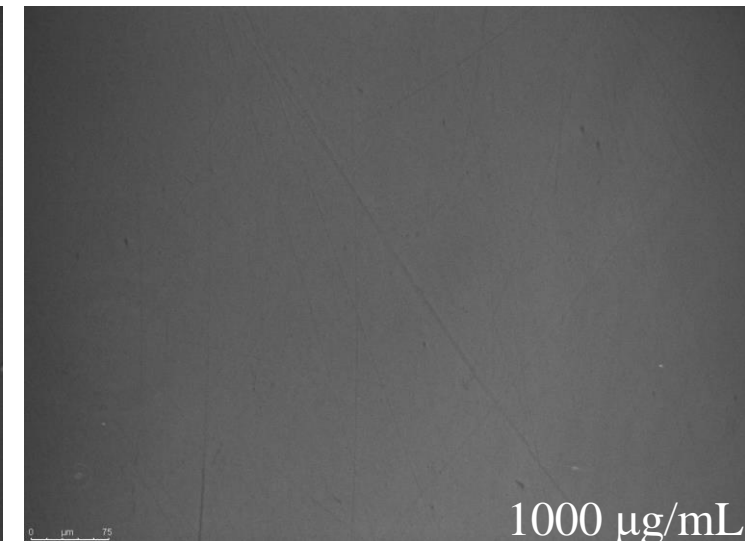

Fig. 3B

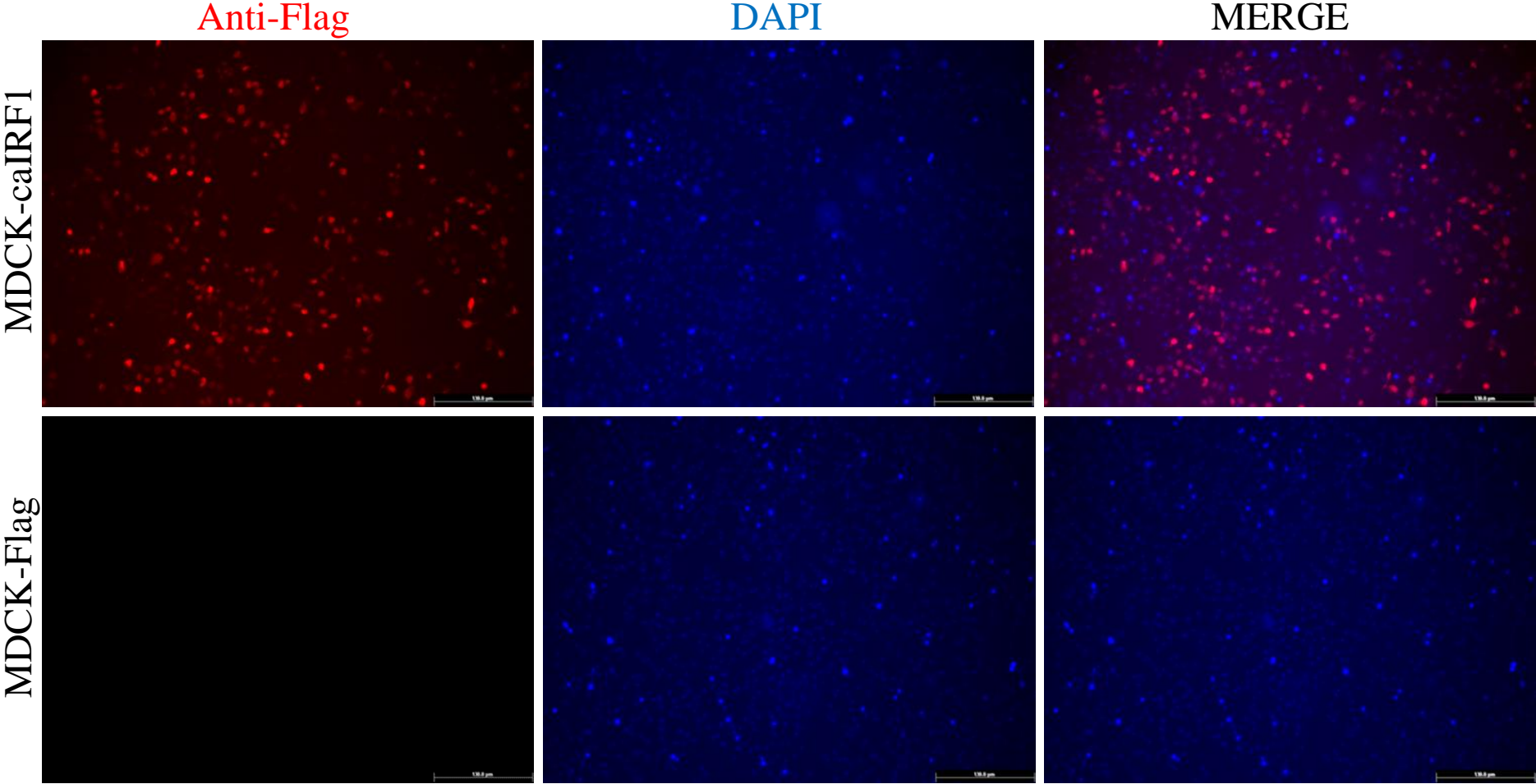

Fig. 3C

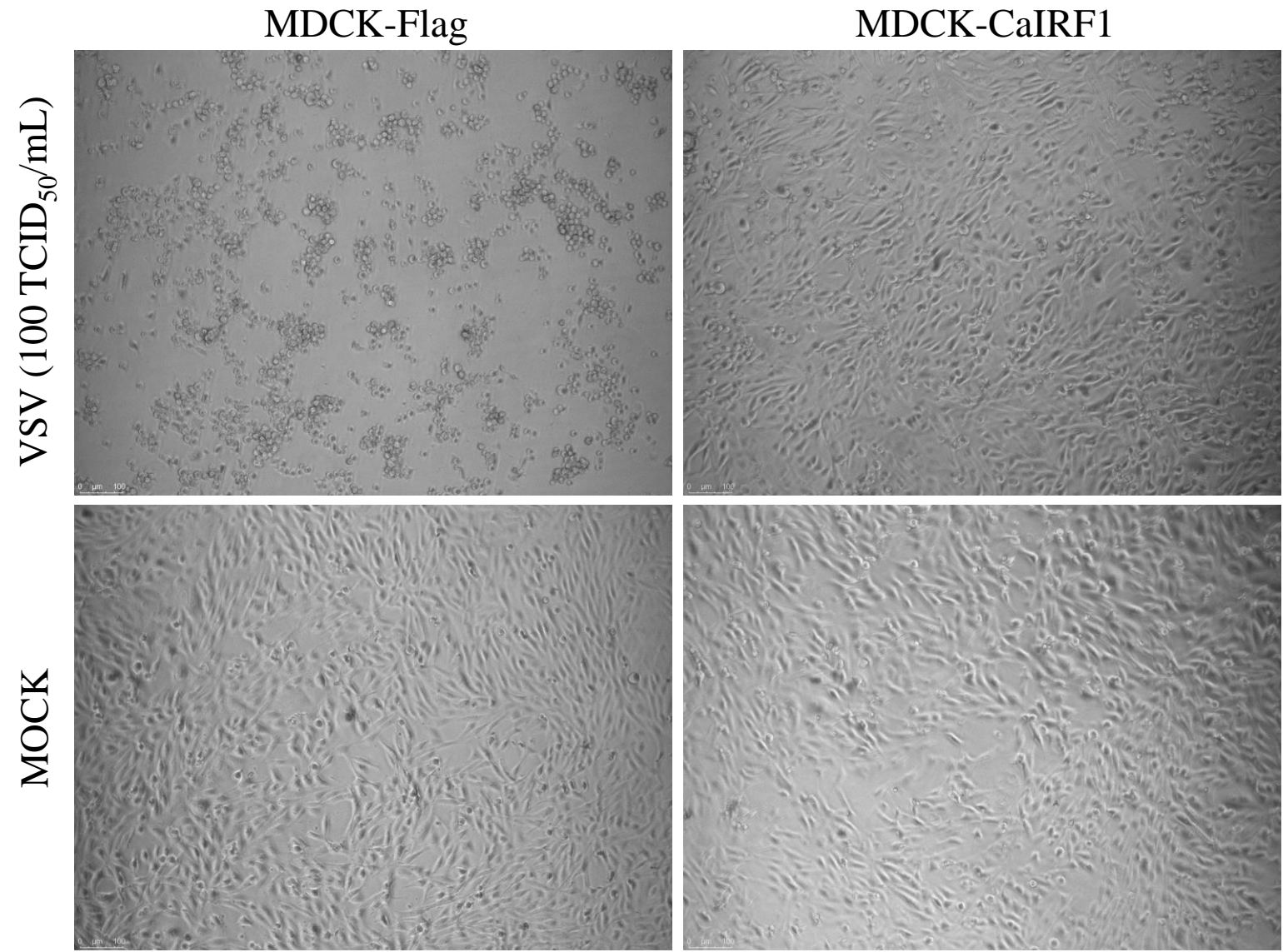

Supplement: Supplementary file 2 — Additional file 2. [file 12917_2022_3539_MOESM2_ESM.pdf]
